# A System of Human Anatomy, General and Special

**Published:** 1858-11

**Authors:** 


ART. VI.—*A System of Humnn Anatomy, General and Special.* By ERASMUS WILSON, F. R. S., Author of "The Dissector's Manual," "A Treatise on Diseases of the Skin," etc., etc. A new and improved American, from an enlarged London edition. Edited by WILLIAM H. GOBRECHT, M. D., Professor of Anatomy in the Philadelphia College of Medicine; Fellow of the College of Physicians of Philadelphia, etc. With three hundred and ninety-seven Illustrations on Wood. Philadelphia: BLANCHARD & LEA. 1858.

With reverence we carefully take from our shelves our venerable copy of Wilson's Anatomy, the only one which has been spared to us, and which bears on its title page the date, 1847. The grimmed cover, changed from its original hue to a shining, greasy black, bears witness to the vicissitudes which it has experienced. The little shavings made by the scalpel, only relieve the venerable shade to which we have before referred. Honest old friend you have served us (and students without number) well, and you will now be permitted to pass the rest of your days in honorable retirement, "on the shelf," to make way for the production of 1858!

Wilson's Anatomy needs no praise. It is in the possession of nearly every doctor and student in the country, having been received here with more favor there any similar work. The last edition is brought out under the direction of Dr. Gobrecht of the Philadelphia College of Medicine. The illustrations have been increased from two hundred and thirty-three to three hundred and ninety-seven, the work itself having been revised and enlarged, containing the same number of pages, but of a larger size than before, and in smaller type. The mechanical execution is excellent, and we think superior to any of the editions before issued by the publishers.

